# Single-procedure 8Fr rheolytic pharmacomechanical thrombectomy for treatment of acute iliofemoral deep venous thrombosis

**DOI:** 10.1186/s42155-024-00447-5

**Published:** 2024-04-02

**Authors:** Nicholas Xiao, Matthew Genet, Rocio Marquez Karry, Elias Hohlastos, Jennifer Karp, Kush Desai

**Affiliations:** 1 Department of Radiology, Division of Interventional Radiology, Northwestern University, Chicago, IL USA; 2 Department of Radiology, Northwestern University Feinberg School of Medicine, Chicago, IL USA

**Keywords:** Deep venous thrombosis, Venous, Thrombectomy, Thrombolysis, Catheter-directed

## Abstract

**Purpose:**

We hypothesize that single-procedure venous-specific rheolytic thrombectomy for treatment of acute iliofemoral deep venous thrombosis (DVT) will result in improved clinical symptoms as measured by the venous clinical severity score (VCSS), as well as durable venous patency, with decreased hemorrhagic risks and costs associated with conventional catheter-directed therapy and prolonged lytic exposure.

**Materials and methods:**

Thirty-three consecutive patients with symptomatic, unilateral, iliofemoral DVT who were treated with single-procedure therapy using the 8Fr rheolytic thrombectomy catheter were retrospectively analyzed from 2012–2021. Abstracted data included technical success (> 95% clearance of acute thrombus), adverse events (AEs), and clinical and imaging outcomes at 1-month and 1-year.

**Results:**

Technical success was achieved in all 33 patients. Mean pre-procedure VCSS was 7.5 with mean edema and pain sub-scores of 2.6 and 1.8, respectively. Post-procedural total mean VCSS at one month was significantly improved (mean post-procedure VCSS = 0.3, mean reduction of 7.2, *P* < 0.01). Clinical improvement was sustained at 1-year (mean total VCSS = 0.2, *P* < 0.01). Primary patency was achieved in all patients at 1-month and 30 (91%) patients at 1-year. Among the 3 patients in which primary patency was not achieved at 1-year, primary-assisted patency was achieved in 2 patients. Secondary patency was achieved in the remaining patient at 1-year. No hemorrhagic AEs occurred in this study.

**Conclusion:**

This study suggests that single-procedure venous-specific rheolytic thrombectomy for treatment of acute iliofemoral DVT is safe and effective, resulting in durable clinical and radiographic results at one year, while also limiting hemorrhagic risks, mitigating costs of admission, and expediting patient discharge.

## Introduction

Current standards of care for deep venous thrombosis (DVT) center on treatment with anticoagulation alone; however, a significant number of patients, particularly those with iliofemoral extension of acute thrombus, develop clinically significant post-thrombotic syndrome (PTS) despite optimal medical therapy.

Sub-analysis data from a large randomized controlled trial (Acute Venous Thrombosis: Thrombus Removal with Adjunctive Catheter-Directed Thrombolysis (ATTRACT)) demonstrated reduction in severity of moderate/severe and severe-grade post-thrombotic syndrome and improvement in venous disease-specific quality of life score in the short term when pharmacomechanical thrombolysis/thrombectomy (PMT) was employed in treatment of acute iliofemoral DVT, compared to anticoagulation alone [1].

Techniques used in catheter directed therapy (CDT) and particularly PMT treatment of acute iliofemoral DVT are non-standardized and vary greatly from operator-to-operator [2–4]. CDT involves use of a multi-side hole infusion catheter for administration of alteplase (tPa) with marked variance in the duration and dosage of lytic [5]. While effective, this technique potentially requires overnight to multi-day admission to an intensive care unit (ICU) level of care for frequent monitoring. This is associated with high costs and resource use related to ICU care, as well as potentially higher risk of hemorrhagic adverse events associated with prolonged exposure to lytic agents [6]. PMT offers the notional benefit of downstream reduction in PTS severity with truncation of treatment time, length of stay, and potentially reduction of the risk of adverse events.

Rheolysis is a PMT approach that consists of the application of high pressure saline spray, with or without physician-directed therapies such as fibrinolytics, directly into thrombus. Via the Bernoulli effect, a low pressure zone is created, resulting in passive aspiration of macerated thrombus. This technology was applied in the ATTRACT trial, with subanalysis performed on “AngioJet-first” therapy. However, the devices used in ATTRACT were not specifically designed for use within the venous system [7]. The 8Fr AngioJet ZelanteDVT system (Boston Scientific, Marlborough, MA) device is a larger profile device which was engineered specifically for treatment of DVT, with the intent of permitting single-procedure treatment without subsequent CDT. However, there remains limited data informing on the durability of its efficacy when used in single-session therapy for acute DVT. In prior studies utilizing other devices, single-session therapy without extended lytic therapy could result in residual thrombus and higher rates of re-thrombosis and re-intervention [8]. The possibility of effective and durable single-session therapy limits the need for ICU level care, limiting exposure to thrombolytics, and potentially hastening improvement in early DVT-related symptoms and patient discharge. The purpose of this study is to assess single-procedure ZelanteDVT-assisted thrombectomy of acute iliofemoral DVT. We hypothesize that this approach is a safe and efficacious technique which results in durable outcomes measured up to one-year post-intervention, while also avoiding potential hemorrhagic risks and costs associated with conventional CDT and prolonged lytic exposure requiring ICU monitoring.

## Materials and methods

**Fig. 1:**
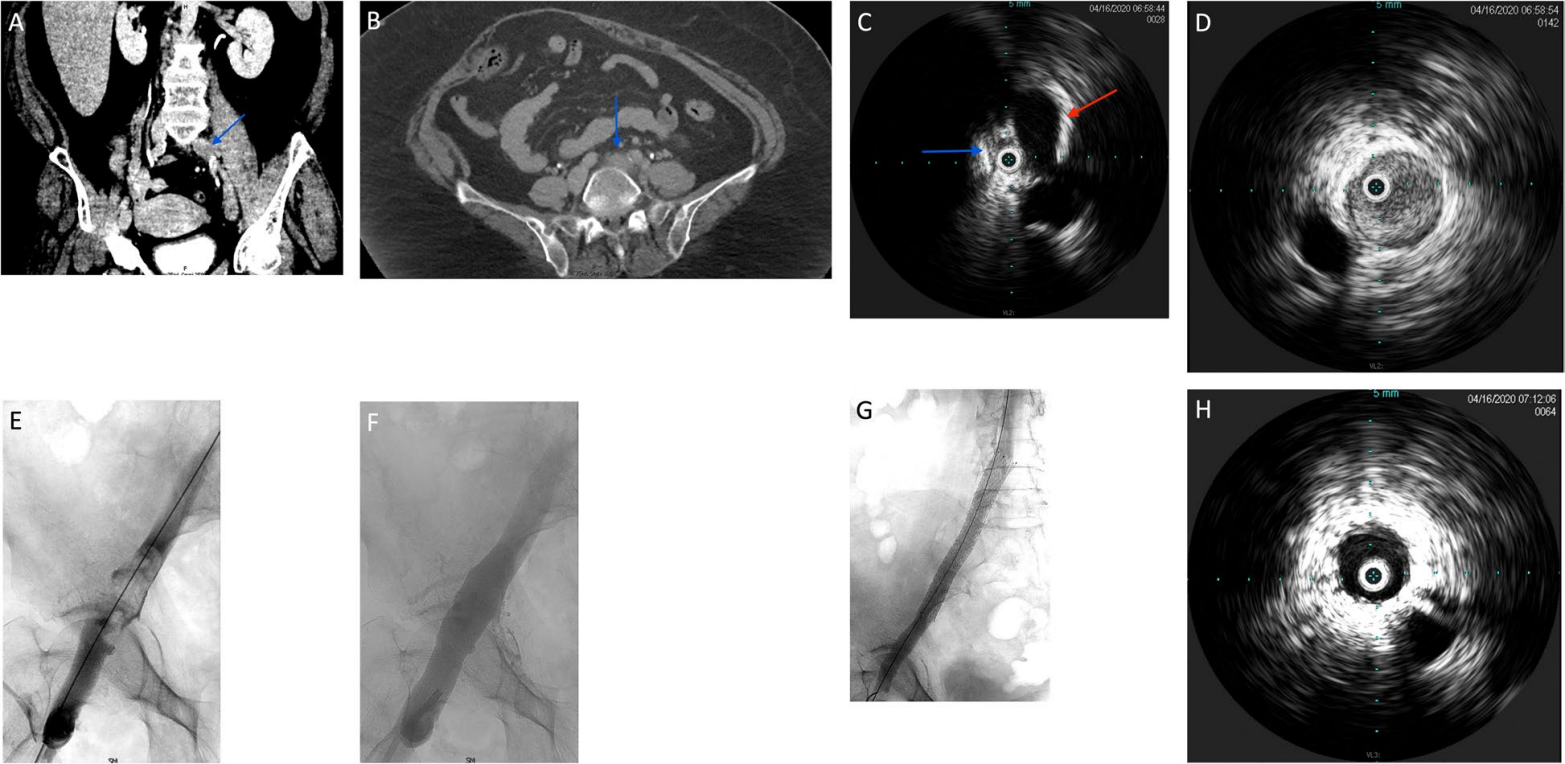
63-year-old woman presenting with acute left lower extremity swelling and pain. **A** Coronal contrast-enhanced CT demonstrates acute, completely occlusive thrombus extending from the proximal left common iliac vein (blue arrow) through the common femoral vein. **B** Axial contrast-enhanced CT at the level of the proximal left common iliac vein demonstrates fat-stranding (blue arrow) and expansile thrombus, suggestive of acute deep venous thrombosis. **C** Intra-vascular ultrasound of the left common iliac vein (blue arrow) demonstrates completely occlusive thrombus and marked luminal narrowing secondary to compression from the crossing right common iliac artery (red arrow), compatible with a May-Thurner compression lesion. **D** Pre-intervention IVUS of the left common iliac vein demonstrates completely occlusive, expansile acute thrombus. **E** Venography of the left iliofemoral veins demonstrate multiple filling defects compatible with acute thrombus. **F** Venography following treatment with the ZelanteDVT system demonstrates technically successful thrombectomy with no residual thrombus. **G** Completion venography following stent placement demonstrates resolution of the compressive lesion at the left common iliac vein and brisk in-line venous flow through the iliofemoral veins without evidence of residual thrombus. **H** Completion IVUS following stent placement demonstrates complete restoration of luminal size without residual thrombus or narrowing

This study was approved by the institutional review board. Thirty-three consecutive patients were retrospectively analyzed from 01/01/2012 to 01/01/2021. Inclusion criteria included those with symptomatic (for 14 days or less), unilateral, iliofemoral deep venous thrombosis who were treated with single-procedure therapy using the 8Fr rheolytic thrombectomy catheter. Exclusion criteria included any patients that demonstrated a contraindication to anticoagulation. Iliofemoral DVT was defined as thrombosis at minimum involving the common femoral vein and/or iliac veins, with involvement of ipsilateral femoropopliteal and tibioperoneal segments permitted. Involvement of the inferior vena cava to any significant degree (defined as over 2 cm) resulted in exclusion from this analysis. The extent of thrombus was determined on pre-intervention imaging using computed tomographic venography (CTV) for the iliac veins and venous duplex sonography for the infrainguinal deep veins (Fig. 1A, B). Patients were excluded if they had any contraindications to anticoagulation.

Access was achieved under sonographic guidance via an ipsilateral popliteal fossa approach (either tibial, small saphenous, or popliteal vein), followed by venography and intravascular ultrasound (8.5Fr Visions, Philips, San Diego, CA) used to assess the extent of thrombus and identify the presence of any compressive lesions (Fig. 1C, D).

The 8Fr rheolytic pharmacomechanical device (ZelanteDVT) was used as the primary thrombectomy device (Fig. 1E, F). Alteplase (tissue plasminogen activator [tPA]. Genentech, South San Francisco, CA) was administered to the thrombosed segments via the “power pulse” mode of the device, and following approximately 30 min dwell time, rheolytic thrombectomy was carried out. Adjunctive devices, including Fogarty embolectomy balloons (Edwards Lifesciences, Irvine, CA), angioplasty balloons and stents were employed as indicated at the discretion of the interventionalist. Additional aspiration or Fogarty thrombectomy was use din cases where there remained wall-adherent material that was not cleared venographically after two passes of the ZelanteDVT device. Stent placement was employed in cases in which there remained a residual lesion following thrombectomy and angioplasty, as determined by venographic or intra-vascular ultrasound findings. Stent selection was determined by the operator and availability of devices at the time of the procedure and was used to treat underlying lesions (Fig. 1G, H). At the time of consultation, Lovenox was recommended as an anticoagulation agent for management of DVT. On the day of the procedure, patients were loaded with Plavix (300 mg). Following the procedure, all patients were maintained on Lovenox for 1 month, and clopidogrel for 3 months. After this time period, all patients were maintained on 81 mg of Aspirin thereafter.

Abstracted data for each patient included procedural details, technical success (defined as a single procedure for successful DVT thrombectomy with greater than 95% acute thrombus clearance), tPA dosage, procedure-related adverse events (AE), as well as clinical and imaging outcomes at 1 month and 1 year [9]. Adverse events were classified according to the SIR adverse events standards of practice [10]. Clinical outcomes were measured via the venous clinical severity score (VCSS) [11, 12]. A paired t-test was used to assess for statistical improvement in VCSS. Significance was accepted at *P* < 0.05. Analyses was conducted using STATA/SE software (version 14.2; StataCorp, College Station, TX). Imaging and technical outcomes were assessed using CTV and venous duplex sonography for evaluation of recurrence of thrombosis and treated vessel patency, measured as primary, primary assisted, and secondary patency.

## Results

Ten males and 23 females were encountered with a mean age of 51 years (Table 1). Technical success was achieved in all 33 patients. Mean intra-procedural tPA dose was 12 mg (range 8–50, SD: 7.2 mg). Mean procedure time was 112.9 min (Range 35–205, SD: 35.9 min). Mean fluoroscopy time was 20.7 (SD: 6.9) minutes and mean radiation dose was 573 mGy (SD: 579). The mean duration of available clinical and imaging follow-up was 1.9 years. Sixteen (70%) of patients presented with provoked DVT. Mean duration of hospitalization following the index operation was 2.42 days (range 1–13 days).

**Table 1:** Patient demographics and technical summary of patients undergoing single-procedure, 8Fr rheolytic pharmacomechanical iliofemoral deep venous thrombectomy

**Patient demographics**	
Total number of patients	33
Male	10 (30%)
Female	23 (70%)
Age (mean, years)	51
Location of DVT	
IVC through femoral vein	8 (24%)
Common iliac through femoral vein	23(70%)
External iliac through femoral vein	1 (3%)
Isolated femoral vein	1 (3%)
**Technical Summary**	
Technical Success	33/33 (100%)
Intraprocedural tPa dose (mg, mean, median)	12, 10
Fluoroscopy time (mean, mins)	20.7
Procedure time (mean, mins)	113
Venous stent placement	28 (85%)
SMART	13 (46%)
Vici	9 (32%)
Venovo	4 (14%)
Zilver Vena	1 (4%)
Abre Venous Stent	1 (4%)
Additional Techniques (no. required)	18 (55%)
6F Envoy catheter for suction thrombectomy	6 (18%)
Angled catheter for suction thrombectomy	6 (18%)
Fogarty balloon mechanical thrombectomy	8 (24%)
Adverse events (AE)	
SIR Major AE	0 (0%)
SIR Minor AE	1 (3%)
Procedure-related hemorrhage	0 (0%)

Stent placement was required in 28 of 33 patients. Thirteen SMART (Cordis, Hialeah, FL), 9 Vici (Boston Scientific, Marlborough, MA), 4 Venovo (Becton, Dickinson and Company, Franklin Lakes, NJ), 1 Zilver Vena (Cook Medical, Bloomington, IN) and 1 Abre venous stents (Medtronic, Warsaw, IN) were deployed. Additional techniques were required in 18 of 33 patients. A 6F Envoy catheter was used for additional aspiration thrombectomy in 6 (18%) of patients and an angeled catheter was used for suction thrombectomy in an additional 6 (18%) of patients. Fogarty balloon thrombectomy was used in 8 (24%) of patients.

**Fig. 2:**
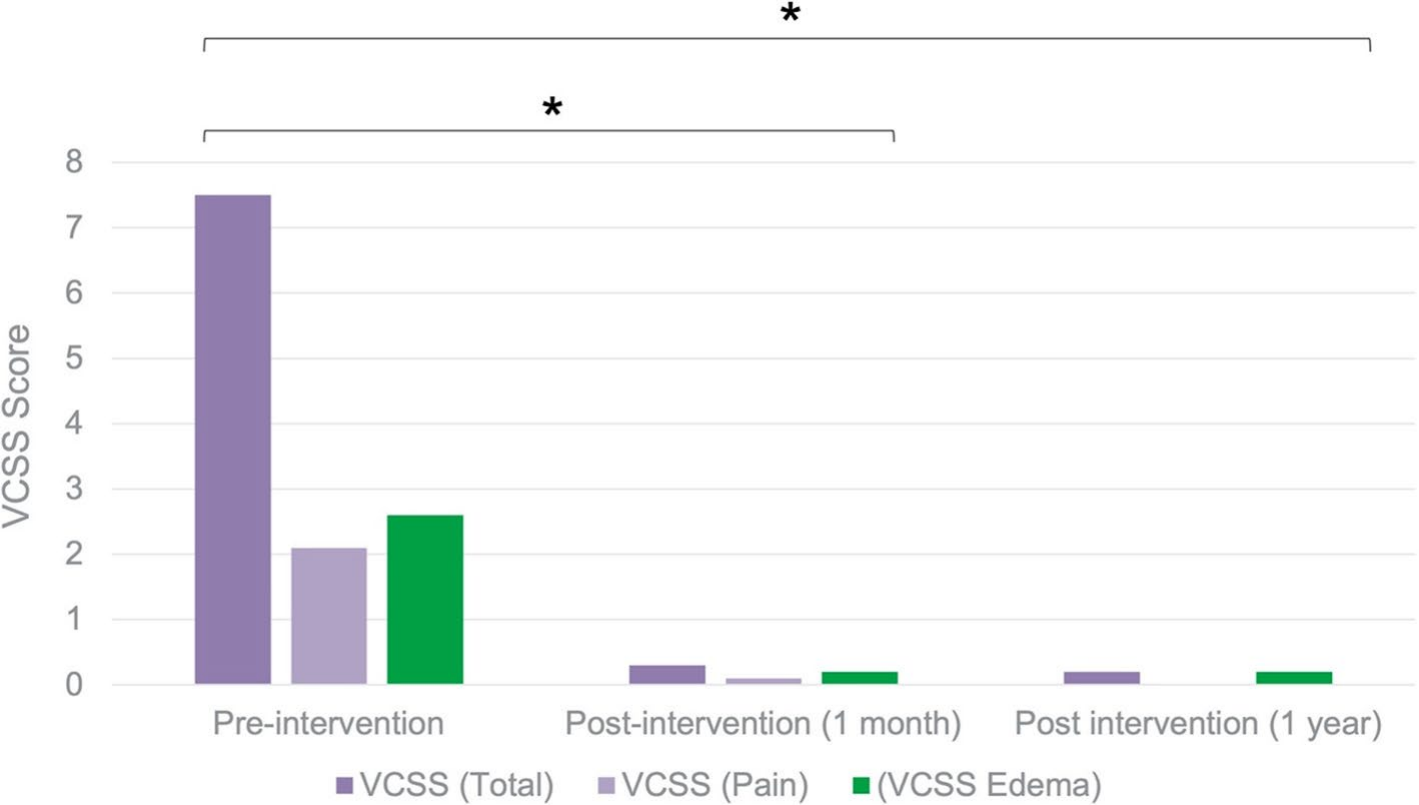
Pre-intervention and post-intervention venous clinical severity scores (VCSS) following single-procedure, 8Fr rheolytic pharmacomechanical iliofemoral deep venous thrombectomy at 1 month and 1 year

Mean pre-intervention VCSS was 7.5 (range 4–11, SD: 1.6) with mean edema and pain sub-scores of 2.6 (range 1–3) and 1.8 (range 0–3) respectively. There was a statistically significant improvement in post-procedural total mean VCSS, with a mean reduction of 7.2 (mean post-procedure VCSS = 0.3, SD: 0.7; 95%CI of VCSS reduction: 6.61–7.82, *P* < 0.01). Clinical improvement was sustained at 1 year (mean total VCSS = 0.2, SD: 1.1; 95%CI of VCSS reduction 6.65–7.91, *P* < 0.01). No patients reported a VCSS >/= 4 at one month (Fig. 2).

Primary patency was achieved in all 33 (100%) patients at 1 month and 30 (91%) patients at 1 year. Among the 3 patients in which primary patency was not achieved at 1 year-follow up, primary-assisted patency was achieved in 2 patients at 1 year. Secondary patency was achieved in the remaining patient at 1 year. No cause was identified for failure to achieve primary patency in these three patients.

Four patients had chronic kidney disease (CKD) at baseline. One AE occurred when a patient with CKD developed acute kidney injury which resolved with nominal therapy on post-operative day 5. No other procedure-related AE occurred; specifically, no significant post-procedural hemorrhage occurred in this study.

## Discussion

This study demonstrates that single-procedure ZelanteDVT-assisted thrombectomy of acute iliofemoral DVT is safe, feasible and results in durable clinical success and high rates of patency through one-year. Clinically, patients at 1 month and 1 year had near complete resolution of their symptoms as assessed by the VCSS. Primary patency at one year was achieved in 91% of patients; 100% patency was achieved with assistance. No post-procedural hemorrhagic events were encountered, suggesting that the single-session approach with on-table use of fibrinolytic is safe as well. The data presented herein remains consistent with smaller cohort studies in which the ZelanteDVT device has been employed [13].

The ATTRACT trial was a landmark randomized control trial which assessed the use of pharmacomechanical for the treatment of acute proximal DVT [1]. Subset analysis revealed a reduction in moderate/severe and severe-grade PTS and improvement in venous disease-specific quality of life with endovascular treatment of patients with acute iliofemoral DVT and at a minimum of moderate-severe symptoms (Villalta 10) compared to anticoagulation alone [14, 15]. Among the 75 patients in ATTRACT whom underwent the “AngioJet-PCDT” strategy, there was a greater improvement in the Venous Quality of Life score and lower incidence of PTS when compared with anticoagulation alone, however these advantages did not persist at 12 and 24 months. Operators in ATTRACT were limited to between 24 and 30 h of tPA infusion, with mean infusion durations of 22, 20 and 19 h for “infusion-first” therapy, Angiojet (DVX or Solent Proxi catheter), and the Trellis Peripheral Infusion System (Coviden Medtronic, Warsaw, IN) respectfully. While the sample size in the present study is limited, our durable results in all patients at one-year suggests that the single-session approach using the AngioJet ZelanteDVT system may also yield comprehensive and potentially lasting clinical benefits. Further follow-up at 24 months will be needed to evaluate whether these results persist. Overall, these findings support the latest Society of Interventional Radiology guidelines which considers catheter-directed therapy to be an acceptable treatment in select patient with iliofemoral DVT [16].

Major bleeding in prior studies ranged from 1.7–5.2% following CDT and PMT of acute iliofemoral DVT out to 24 months [7, 17]. We observed no AEs related to major hemorrhage in the present study of 33 consecutive patients out to one-year. While a small sample size, the results suggest that decreased fibrinolytic exposure, in this study limited to on-table use only, may result in lower rates of AEs related to iatrogenic bleeding.

Single-procedure venous-specific rheolytic thrombectomy for acute iliofemoral DVT resulted in high patency rates (100%) at one-month, and durable primary patency at one year (91%). This is significantly improved from prior studies demonstrating approximately 50% patency at 1 year employing older rheolytic devices [8].

This study has limitations. This is a single-center study, with most procedures performed by a single, experienced operator, and therefore these technical results may not be readily generalizable. The sample size in this study is also small, limiting detection of rare AEs. Additionally, numerous other thrombectomy devices with various mechanisms of action are now available for endovascular treatment of DVT, and comparison with other devices may further help identify an optimal approach to treatment of these patients. Finally, further research and long-term clinical follow-up will be needed to evaluate for long term clinical success and patency rates.

In conclusion, the findings of this study suggest that single-procedure venous-specific rheolytic thrombectomy for treatment of acute iliofemoral DVT is both safe and effective, resulting in durable clinical and radiographic results at one year, while also limiting hemorrhagic risks, mitigating costs of admission, and expediting patient discharge.

## Data Availability

The data are presented in the main manuscript. There was no data that was withheld within the results of this manuscript. Any additional associated data that could be obtained with further review will be made available upon reasonable request.
